# The provision of person‐centred care for care home residents with stroke: An ethnographic study

**DOI:** 10.1111/hsc.13936

**Published:** 2022-07-23

**Authors:** Eleanor Stevens, Stephanie G. Clarke, Jean Harrington, Jill Manthorpe, Finbarr C. Martin, Catherine Sackley, Christopher McKevitt, Iain J. Marshall, David Wyatt, Charles Wolfe

**Affiliations:** ^1^ School of Life Course & Population Sciences King's College London London UK; ^2^ Guy's and St Thomas' NHS Foundation Trust London UK; ^3^ University Hospitals Birmingham Department of Physiotherapy Birmingham UK; ^4^ National Institute for Health and Care Research (NIHR) Policy Research Unit in Health & Social Care Workforce, King's College London London UK; ^5^ Faculty of Medicine and Health Sciences University of Nottingham Nottingham UK; ^6^ National Institute for Health and Care Research (NIHR) Applied Research Collaboration (ARC) South London London UK; ^7^ National Institute for Health and Care Research (NIHR) Biomedical Research Centre (BRC) Guy's and St Thomas' NHS Foundation Trust and King's College London London UK

**Keywords:** care homes, nursing homes, person‐centred care, qualitative research, residential facilities*, stroke

## Abstract

Care home residents with stroke have higher levels of disability and poorer access to health services than those living in their own homes. We undertook observations and semi‐structured interviews (*n* = 28 participants) with managers, staff, residents who had experienced a stroke and their relatives in four homes in London, England, in 2018/2019. Thematic analysis revealed that residents' needs regarding valued activity and stroke‐specific care and rehabilitation were not always being met. This resulted from an interplay of factors: staff's lack of recognition of stroke and its effects; gaps in skills; time pressures; and the prioritisation of residents' safety. To improve residential care provision and residents' quality of life, care commissioners, regulators and providers may need to re‐examine how care homes balance safety and limits on staff time against residents' valued activity, alongside improving access to specialist healthcare treatment and support.

**What is known about this topic**

- Many stroke survivors living in care homes have severe limitations regarding self‐care.
- Many care home residents living with stroke have limited or no access to stroke specialist care.

**What this paper adds**

- Having had a stroke with associated disability is not perceived by all care home staff to be an important attribute for care planning purposes.
- Meeting needs and preferences, particularly in valued activities, is often not achieved for residents with stroke‐related disability.
- Care home staff have limited access to training and support to help them care optimally for residents with stroke and respond effectively to stroke‐related changes.

## 1 INTRODUCTION

In the United Kingdom (UK), although most stroke survivors return home after hospital treatment (King's College London, 2021a) some will need to move to a care home. An estimated 11% (United States [US]) to 18% (UK) of care home residents aged ≥65 years have a stroke diagnosis (Caffrey & Sengupta, 2014; Shah et al., 2011). We use ‘care home’ to include both care homes with a registered nurse on site and those without. Stroke survivors who move to care homes typically have severe limitations in daily self‐care, including mobility, grooming, going to the toilet, dressing and eating (Clery et al., 2020; Dutta et al., 2018). The cohort moving to care homes from their own homes later on after a stroke has not been characterised; dementia and increasing disability are likely to contribute to deciding to move.

UK stroke care guidelines recommend that care home residents should receive the same standard of treatment/appropriate equipment as those in their own homes (Intercollegiate Stroke Working Party, 2016). The guidelines recommend training care home staff in stroke effects (physical, cognitive/communication, psychological, social) and managing common activity limitations. In practice, stroke‐specific training is not mandatory for these staff. International reviews of stroke care in residential facilities describe inadequate and unequal access to rehabilitation therapists and equipment, stroke‐specific care (formal reviews, health service contacts, nursing skills) and secondary stroke prevention (Gonçalves‐Bradley et al., 2015; Teo & Slark, 2016).

‘Person‐centred care’ describes approaches where care providers work in partnership with the care recipient, supporting informed choice in care and treatment, and make reasonable adjustments to meet individual needs and preferences. The concept of person‐centred care has been used in research (Brownie & Nancarrow, 2013) and social care policy: for instance, it forms part of the regulation of UK care homes (Health and Social Care Act 2008 [Regulated Activities] Regulations 2014). However, research has highlighted a knowledge/practice gap among care home staff regarding the concept (Güney et al., 2021).

Research on residents with stroke is limited (Teo & Slark, 2016). The main objective of this present study was to understand perceptions and experiences (of staff, affected residents, their relatives) of the needs and care of residents with stroke to better comprehend how their care and quality of life might be improved. While it is reasonable to think that ‘person‐centred’ care should be sensitive to stroke‐related disability, residents and staff may not prioritise stroke‐specific care needs, given that most residents have multiple long‐term conditions (Gordon et al., 2014). Therefore, we also sought to understand the relevance of stroke to care in perception and practice.

## 2 METHODS

We undertook semi‐structured interviews and overt observation (Atkinson & Hammersley, 1998) in care homes located in socio‐demographically diverse urban South London, England. The methodology was chosen to reveal the reality of care practices and lived experiences (Hammersley & Atkinson, 2007).

### 2.1 Sampling and Recruitment

#### 2.1.1 Care homes

We aimed to recruit six diverse homes (based on resident number, ownership [independent/corporate group], registration/specialism, Care Quality Commission [CQC, England's independent health and social care regulator] rating) (Table 1). We used South London Stroke Register data to identify homes with resident stroke survivors (King's College London, 2021b). Fieldwork took place in four homes during 2018/2019; subsequently data collection ended due to the COVID‐19 pandemic.

**TABLE 1:** Characteristics of participating care homes and numbers of participating individuals at each care home

Care home	Resident number	Facilities	Care home registration	Specialism	Ownership	Care quality commission rating—range Outstanding, Good, Requires Improvement or Inadequate	Residents' funding source	Participant number
1	48	4 resident floors. Recent part refurbishment of communal areas	Care Home	Dementia	Not‐for‐profit national company	Good	Majority publicly funded	2 residents, 1 relative, 4 manager/staff
2	51	3 resident floors. Unused activity space. Undergoing modernisation	Care Home with nursing	Dementia	For‐profit regional company	Requires Improvement	Majority self‐funded	2 residents, 1 relative, 3 manager/staff
3	70	4 resident floors	Dual registered	Dementia	For‐profit regional company	Good	Majority publicly funded	2 residents, 1 relative, 6 manager/staff
4	63	3 resident floors around a courtyard. In need of modernisation	Care Home with nursing	Dementia Physical disabilities Sensory impairments	For‐profit	Requires Improvement	Majority publicly funded	2 residents, 1 relative, 3 manager/staff

#### 2.1.2 Participants

Following agreement with home managers, researchers invited staff to participate, using convenience sampling based on a range of roles (Table 2).

**TABLE 2:** Participant types and numbers

Participant type (code prefix for quotes/excerpts)	Participant number (of which, number interviewed)
Resident with stroke (Res)	8^a^ (4)
Relative of resident (Fam)	4 (4)
Registered manager (Man)	4 (4)
Senior administrator (Adm)	1 (1)
Senior nurse (SNu)	1 (1)
Nurse (Nu)	3 (3)
Senior care worker (SCW)	3 (3)
Care worker (CW)	2 (2)
Activity coordinator (AC)	1 (1)
Physiotherapist employed by home (Ph)	1 (1)
Total	28 (24)

^a^ Eight residents consented (or their consultee gave favourable opinion) to participate. Of these, three were unable to participate in an interview, and one declined an interview.

We sought to include all residents with stroke in the observation element. Our protocol excluded from interview residents with severe communication or memory problems. In these instances, we sought to interview a relative.

Home managers identified residents with a recorded stroke diagnosis (Table 2). If an unrecorded stroke was suspected, (where possible) researchers spoke with relatives to confirm. Staff asked eligible residents if they wished to speak with a researcher about the study.

#### 2.1.3 Ethics

We gave potential participants an information sheet, allowing 24 h (minimum) before seeking written consent. During interviews and observations, if participants appeared distressed or uncomfortable, the researcher changed topic or postponed/ended data collection. No personal care or other sensitive activities were observed.

If the researcher judged that a resident did not have capacity to consent to an interview or observations, we sought advice on their participation from a consultee following the requirements of the Mental Capacity Act (Mental Capacity Act, 2005). In practice, we identified *personal* consultees (family/friend) via staff.

We did not record data (directly heard or observed) without consent/consultee opinion. We sought consent from staff members working in participating residents' areas of the care home before observing interactions. Non‐participating residents encountered in communal areas were told about the study and asked if they would prefer researchers to leave. Posters displayed in communal areas provided brief study information, researcher photographs and contact details. The London—Camberwell St Giles Research Ethics Committee (Ref 18/LO/0805) approved the study.

### 2.2 Data collection and analysis

The fieldwork team comprised two social scientists, E.S. and J.H., and a researcher physiotherapist, S.C. When consent/consultee opinion allowed, we used an ethnographic approach, spending time observing, being with and speaking with participating residents and staff (we had little opportunity to observe managers). We held introductory ‘off the record’ conversations with managers, staff and residents during an initial familiarisation period. Interviews lasted 30 min on average, were audio‐recorded and transcribed verbatim (see Supporting information S1: topic guides). We used unstructured observation to record daily life for residents with stroke. During our fieldwork, this mainly comprised routine events such as mealtimes, moving around the home, TV‐watching, and interactions with staff and visitors. Each home was visited five/six times covering different days of the week and times of the waking day (approximately 12 h of observation per home). Handwritten field notes were typed up as soon as practicable after each visit.

Transcriptions and observation notes (details anonymised) were stored securely. Thematic data analysis (Braun & Clarke, 2006) used both software (Nvivo v11 QSR International Pty Ltd) and paper‐based methods (theme concept maps). Transcripts/observation notes were coded as a single dataset. First, researchers independently read and coded the data descriptively and inductively (i.e. not conforming to pre‐specified codes, allowing ‘unexpected’ themes to develop) (Sandelowski, 2000). Second, the researchers and supervisor (C.M.) discussed code interpretations and their relevance to the research questions to develop a code categories framework (themes and sub‐themes) (Timmermans & Tavory, 2012). The framework enabled material from observations and interviews to be grouped for comparison within and across themes and care homes. Similarities and divergences – stated practice/attitudes versus observed practice, and one source versus another – were noted in ‘memos’ which linked data extracts within themes. Themes and underlying data were discussed with all authors who included care home experts, clinical specialists in primary care, geriatric medicine and physiotherapy and health services researchers.

## 3 FINDINGS

Three of the four participating homes provided nursing care alongside personal care and all specialised in caring for people with dementia (Table 1). They were similar in layout: ground floor reception area; keycode access to floors where residents with more complex needs had their bedrooms; individual *ensuite* bedrooms along corridors; communal spaces (seating area with TV/radio, dining area); kitchens (staff use only, on floors where we observed residents) and offices. Nursing care was provided on specific floors. We undertook 21 interviews (24 participants [three paired interviews]) and observations (28 participants) including managers, staff, residents and visiting relatives (Table 2). Staff had a range of job roles and grades: managers, (senior) care worker, (senior) nurse, activity coordinator, physiotherapist and administrator. Interviewed residents had lived in their home for between 2 and 8 years (approximately). Three participants (one in each of three homes) lived in nurse‐led care areas of the home.

We organised our findings into five main themes, incorporating data from both interviews and observations and evidence from all homes. We have omitted any home identifier to ensure participants' anonymity and specify where an observation relates to some rather than all homes. We use ‘staff’ to mean home staff in general.

### 3.1 Limited stroke awareness

#### 3.1.1 Formal and informal training

Care workers reported not having undertaken training in caring for people with stroke specifically (or other condition‐specific training, except on dementia: such training is not mandatory for social care staff in England). They unsurprisingly reported skills gaps in various aspects of care, including secondary prevention, and knowing which professionals to contact about concerns. A manager explained how staff might receive ‘indirect training’ (Man02) from clinical specialists via their input into care plans. However, opportunities to improve knowledge/skills were not always taken up. One staff member (CW01) working in a non‐professional role, but with more than 20 years of professional nursing experience (including time specialising in stroke), had offered to train colleagues, but this had not been taken up.

#### 3.1.2 Awareness of residents' stroke diagnosis

We found nursing and non‐qualified staff had incomplete awareness of whether residents were stroke survivors; one nurse thought there were ‘probably’ (Nu02) residents with prior stroke of which staff were not aware. Indeed, in one home [author 2] confirmed with a relative that a resident was a stroke survivor, yet staff did not recognise the resident as such and reported that their records did not contain this diagnosis. One senior care worker (SCW01) reported that no residents had had a stroke (new or recurrent) in their 20 years of experience, which is improbable.

#### 3.1.3 Knowledge of stroke prevention, signs and effects

Staff had variable basic knowledge concerning stroke. Those who had witnessed an incident of stroke recognised it as a medical emergency. They had some understanding of stroke effects on mobility, continence and speech, based on resident observations. In general, they were unaware of other common long‐term consequences of stroke, including cognitive and visual impairment and emotional/psychological problems. Risk factor and secondary prevention awareness was also low. Managers also reported receiving little relevant information from the discharging hospital regarding care needs of a new resident who had been treated for stroke.

A nurse raised the potential to notice change in a resident which might indicate recurrent stroke:

…there were days when [residents with stroke] have unusual behaviour, maybe they get confused or they have different movements […] [they can] develop a stroke any time again […] So we learned to be aware of that […] (Nu02)

Nurses reported that changes in residents' health status or behaviour were recorded in daily care documentation and discussed at clinical risk meetings.

A senior care worker implied that continuity of staff was important in recognising any such change (SCW01):

…it's very seldom that I'm off two days in a row […] [so] I'll be able to say 'oh [resident] just doesn't seem right today.' (SCW01)

### 3.2 Sensitivity to stroke‐specific needs

‘Stroke’ was rarely used descriptively in daily caregiving. One senior nurse described their home's lack of prior consideration of stroke‐related care:

…there is no specialities when it comes to delivering care. […] I don't know why […], we've not picked on that to say OK let's sit down and do something about stroke. (SNu01)

Managers however perceived a need for awareness of a resident's disabilities, including stroke‐related disability, to deliver ‘person‐centred’ care:

…if they had a stroke maybe they need a bit more support, because they have parts of their body that are weaker […] but I don't think that it's going to be something specific […] for stroke survivors. (Man03)

[staff would say to the general practitioner, GP] ‘[resident with stroke] has swallowing difficulties and you've given him all tablets so could you change it to liquids’ […] that's what person‐centred care is all about. (Man02)

Similarly, a nurse conveyed the importance of early therapeutic intervention after stroke:

…if [upper limb rehabilitation] not being done in the beginning, it might be too late. […] [Muscles] become very quickly contracted (Nu01)

Staff two homes reported that colleagues lacked disability awareness. One care worker commented that their colleagues needed to be more ‘aware of what they are doing with a patient with stroke’ (CW01), that is, sensitivity to one side of the body being affected. Similarly, a physiotherapist commented on the need for specific staff training to avoid injury when assisting residents with stroke‐related impairment:

Especially with stroke, you have to treat them a bit different, you have to teach [staff] about the shoulder or the dragging leg. (Ph01)

### 3.3 Met and unmet needs of residents with stroke

#### 3.3.1 Rehabilitation and promoting independence

Some participants expressed a belief that a lack of physical or occupational therapy since moving to the home had led to avoidable deterioration (mobility, upper limb function). Based on participants' comments, interventions or contacts by National Health Service (NHS) community therapy services varied between homes but was generally limited, particularly occupational therapy (two homes employed their own physiotherapists suggesting they were under‐served by community NHS services). Other needs described as not fully met included psychological support to adjust to disability, and support to identify accessible activities.

In general, staff conveyed an understanding of the importance of residents being permitted or enabled to do things for themselves. For example, one senior care worker said, ‘we try to let them be as independent as they possibly can for as long as they can’ (SCW02). This perhaps indicates the influence of dementia training and awareness. However, another staff member perceived that the lives of residents with stroke‐related disability were inherently limited:

…they are either bed‐bound or chair‐bound, so there is nothing much to do with them [other] than to have a good talk. (SNu01)

Relatives commented on a lack of rehabilitative activity or encouragement to attempt self‐care (Fam02, Fam04). A care worker (CW01) mentioned that residents with upper limb impairment did not have adapted cutlery; it appeared that the home staff had not considered providing this. Another relative (Fam03) described how the home's physiotherapist had to persuade other staff to support transfers from bed to chair for their parent. This had ‘taken a while’ (Fam03) to be normalised. The resident in question described staff as being ‘very cautious’ (Res03). Another relative reported that their parent had developed hand contractures: their hand splint had not been used (Fam04).

#### 3.3.2 Participation in planned activities

We observed or heard of examples of staff supporting residents with stroke, who had limited mobility, to participate in activities delivered by staff or others: chair‐based exercise, ‘music and movement’ (Res08), taking a resident to a concert (Fam03) or providing art materials:

[Resident with stroke] loves her colouring so we can bring her paints and if we're doing arts or crafts […], [staff] will bring the stuff into her room. (Man01)

However, some planned activities were not differentiated by ability or inclusive. Discussing a recent activity (beanbag throwing/catching) a resident said:

…it's all right for everybody else that hasn't had a stroke, I can't do what other people are doing. (Res08)

One relative perceived that differentiation must be too ‘difficult’ (Fam04) for staff to manage. An activity coordinator (AC01) had a different perspective, reporting a perception among colleagues that leisure activities were unimportant (compared to care tasks). Supporting this, we observed occasions when residents were not invited to attend activities taking place on a different floor of that home. Another home's ‘activity space’ was used for storage.

#### 3.3.3 Participation and autonomy in valued activity

Residents with stroke said they valued purposeful activities, similar to those in which they used to engage. For example, one resident, a keen library user, was asked about their aspirations for daily life:

Very limited really. I would like to walk to some of the places we take the wheelchair to […]. To the library, a long distance to go. (Res03)

Another resident was reported to enjoy purchasing items from the home's ‘pop‐up shop’ for themself and for others unable to access it.

An instance of staff engaging with a resident's valued activity was recorded in another fieldnote:

Music is being played in the lounge. [Res06] stands up with a hand on their walking aid. A care worker comes over. She begins dancing, taking hold of [resident]’s hand to prompt them to join in, which they do with a smile. [Res06] tells her ‘You are a sister to me now, not a friend’. (Observation excerpt, Res06)

Although managers and staff often referred to the importance of person‐centred care, residents' interests and preferences were not always supported. Overall, activity in the homes revolved around care routines and staff availability. One resident (Res01) (a wheelchair user) complained they had no opportunity to do any gardening tasks in the home's garden. Another expressed sadness when describing aspects of life in the home, such as absence of choice in ‘bedtime’ and lack of meaningful social interaction. A fieldnote captured them voicing how their mood was affected by being helped to walk to the toilet instead of being wheeled:

[Resident] explains that if certain staff are on duty they support [resident] under their ‘bad’ (stroke‐affected) arm and [resident] uses their stick to walk to the toilet. I ask how that feels. [Resident] says ‘Oh!, it is wonderful! Just to stand is wonderful!’ [Resident] has a catch in their voice. (Observation excerpt, Res08)

#### 3.3.4 Staff interactions with residents with stroke‐related communication impairment

We observed and heard of instances when staff enabled residents with communication impairment to express themselves. In one home, staff used communication books and ‘picture menus’ with residents with expressive aphasia.

We occasionally observed (in two homes) staff answering on residents' behalf (regarding preferences, such as whether the resident would like items moved in their room) rather than waiting for a response. One nurse also observed this and commented that staff needed to allow more time for residents with aphasia to communicate:

We're asking and we're answering now ourselves for them and they're supposed to just with their eyes say ‘I agree with what you say’ […] after stroke people, they have [aphasia] […] they try and it's not coming out and [staff say] ‘what you say? What you say?’ and they [resident] stop talking. (Nu01)

A manager reported needing to remind staff that residents' communication impairment should not be confused with lack of broader cognitive skills. In doing so, the manager themself expressed a misguided belief that ‘thumbs up’ is sufficient to indicate understanding:

[…] I have to initially keep saying to staff ‘this is not dementia, this is just the person can't talk but try saying hello and try speaking and he'll give you a thumbs up and he'll smile at you, which will tell you he knows exactly what you're talking about’. (Man02)

### 3.4 Time pressures on care

Staff often said limited time and staffing restricted opportunities for rehabilitation and exercise:

…the physio will come and make a plan, like every day ten minutes do this exercise. Never ever seen [staff] stay and do [the exercise] […] They'll say ‘I'm busy’. (Nu02)

One resident reported that staff tended to transfer them by wheelchair to the toilet rather than assisting them to walk during busy times:

I tell [staff] with my stick I can walk with you holding the other side of me […] to the toilet […] 15 steps and 15 steps back […] doesn't take them long. (Res08)

We observed such uses of a wheelchair in one home at especially busy times of day for staff, such as around staff handover and mealtimes.

Staff said administrative duties, such as completing care records, put pressure on time available for caring. A senior care worker expressed frustration that the quantity of mandatory online documentation meant (in their view) excessive time spent away from residents. They felt this risked harm to residents:

[SCW01] is in the dining room finishing off the medication round. [SCW01] tells me they are still trying to cope with completing the CQC paperwork [online]. In frustration they throw down the [medication] paperwork in their hand and tells me tomorrow they will get what they have [i.e. they will submit only as much documentation as they have already done]! They cannot care for residents and do this [documentation]! […] They describe residents not being cleaned promptly and resultant damage to skin, bedsores. To avoid this sort of thing is their priority, but administrative burdens make this very difficult. (Observation excerpt, SCW01)

In two care homes staff used tablet computers to record personal/nursing care tasks and observations. This was supposed to be done at the point of care and promote accurate record‐keeping, but staff commented that on busy shifts records would be completed retrospectively. This practice was also observed in a home that used paper‐based records. Staff shortages increased time pressure:

[SNu01] is seated behind the desk filling in medication forms. They are angry. [SNu01] tells me they were the only [staff member] on the ground floor today – that is why they have to retrospectively fill in the medication. The two [care workers] they were meant to have had not turned up. (Observation excerpt, SNu01)

Managers explained that some residents, including those with stroke, would benefit from one‐to‐one care to support participation in planned and valued activities, but the funding paid by local authorities was insufficient to provide this level of staffing. An activity coordinator identified funding as a key limitation to delivering better care, which they perceived should incorporate activity as well as essential care:

the [care worker] can't interact with everyone and she has so much things to do, paperwork, training, caring. […] If someone really wants to deliver the best care, I think you need to look into getting more funding to activity. (AC01)

### 3.5 Safety and risk

Staff named safety as a priority for care:

Safety. That's the first word that comes to my mind […] ensure [residents] are safe and protected. (Nu03)

So in the first instance, we have safe handling training, which I feel is very important for stroke, post stroke. (Man02)

One relative believed deterioration in their parent's mobility was due to over‐reliance on wheelchair use. The staff perceived that this resident was at risk of falling and judged he was not able to decide for himself how to mobilise. The relative perceived that staff prioritised safety as ‘they do not want to have accidents’ (Fam04) and had limited time:

Well it's for safety isn't it. […] he walked [into the home] […] [now,] they don't get him to walk […] I know [resident] told me it was the same for her. […] she was walking when she came in, and you know she's fully with it [cognitively] […] and they don't let her do it. I think it's probably, they don't have time. It's easier […] [to] wheel them and then stick them there for safety. (Fam04)

Some managers and staff described staff fearing they would be held responsible for adverse incidents, such as injury to a resident, if the care plan or risk assessment was not followed exactly:

Activity coordinator: If we do things on our own [initiative] and then something went wrong it would be [a major problem].

Interviewer: And does that sense of responsibility in regard to safety stop you from doing some things?

Activity coordinator: Yeah […] it's laws and regulations […] they will take you […] it's your fault (AC01)

Although staff told us safety was paramount, in two homes we observed potentially risky practices: post‐stroke residents being assisted to eat or handed a meal while in slumped positions in lounge chairs or recumbent in bed, increasing the risk of food aspiration. This could be due to incomplete staff training, but evident time pressures on staff around mealtimes probably also contributed to them not acting on residents' poor positioning: for example, care workers could not hand out meals and simultaneously safely reposition someone.

The managers of two homes described examples where they would accept greater risks to improve the quality of residents' lives. For example, one (Man02) said residents with capacity to understand the risk to skin integrity could decide to sit out of bed for extended periods. This manager also described having to overcome staff's initial apprehension about introducing self‐service hot meals for some groups of residents as ‘another example of how health and safety was coming in the way of [residents] maintaining their independence’ (Man02).

## 4 DISCUSSION

We report findings from a study using observations and interviews (*n* = 28) including various staff roles/grades, residents and relatives from four care homes. Our analysis identified five main themes: limited stroke awareness among staff, sensitivity to stroke specific needs, met and unmet needs of residents with stroke, time pressures on care, and safety and risk. Some themes represent challenges which may affect residents regardless of health conditions, but others, such as staff skills in positioning residents or anticipating swallowing problems, particularly affect residents with stroke.

This study provides evidence that stroke is under‐recognised by home staff. Some staff lacked disability awareness in their interactions with residents with sensory impairment or a stroke‐affected side. Some staff believed post‐stroke residents were invariably immobile and unable to participate in leisure activity beyond conversation. Although awareness of a stroke diagnosis is arguably not essential to assess current self‐care abilities and plan daily care accordingly, specialist skills and equipment (such as adapted cutlery, not seen in the homes) are required to optimise self‐care participation. Most home staff do not have specialist skills and knowledge (Rigby et al., 2011; Skills for Care, 2020a), which are hard to promote in a sector with extraordinarily high staff turnover (Skills for Care, 2020b), and have few opportunities to learn directly from specialists. Consequently, staff may not recognise deterioration as stroke‐related or understand the potential for someone with stroke to maintain or even improve function and participation.

Managers welcomed clinical specialists' input into care plans, but received little information from discharging hospitals relevant to caring for a specific resident with stroke. Some described difficulties accessing NHS community therapy services. Reviews of healthcare services to care home residents have long described unclear delineations of responsibility within/between homes and wider health and social care systems (Robbins et al., 2013) and emphasised the importance of joint care planning by home staff with visiting clinicians (Goodman et al., 2016).

Home managers and staff generally expressed support for care practices which promoted person‐centred care and residents' independence, and we recorded examples of staff supporting residents' leisure participation. However, during observations in two homes staff often lacked time to even engage in conversation, much less support a library visit (for example). This may be a widespread problem across homes: a survey of English care homes in 2014/2015 found a third of staff were “never or almost never aware of a resident being taken out of the home for their enjoyment”; 15% were never or almost never aware of “an activity planned around a resident's interests” (Cooper et al., 2018).

Residents' preferences for care were not met when staff were too busy to do so safely, particularly residents being moved in a wheelchair instead of being assisted to walk. This occurred even when a resident so valued walking to the toilet that they knew the number of steps needed.

This study has limitations. The COVID‐19 pandemic precluded follow‐up fieldwork in the four featured homes and fieldwork in the planned two further homes. We excluded residents with severe communication/cognitive impairment from interview, limiting their contribution to the study. However, we attempted to interview residents with less severe communication/cognitive impairment using a specially‐designed information sheet. Regarding strengths, our study design allowed us to triangulate data from observations and in‐depth interviews. Based on study team experiences, we believe our findings are likely to be consistent with the range of care home practice and resident experience in the UK at least, although the employment of physiotherapists is not typical.

## 5 CONCLUSIONS

Care home staff need to be supported (with paid time, encouragement) to access guidance and training about the effects/consequences of stroke for the individuals they look after. Care home providers need to have adequate funding for staff training and to employ sufficient staff so that training can be put into practice. Training could include developing understanding of the value of a comprehensive multidisciplinary team assessment of specific impairments experienced by individual stroke survivors—as recommended in stroke guidelines (Intercollegiate Stroke Working Party, 2016; Winstein et al., 2016)—and recommendations/strategies to overcome them in partnership with other professionals.

However, it seems unlikely that the complex changes needed to improve the lives of residents with stroke will be achievable without further effort to overcome systemic problems affecting adult social care (staff working under pressure with insufficient time, high staff turnover and vacancies, low staffing levels, inadequate access to health data and professional support and so on) (Sampson et al., 2020).

More opportunities for valued activity could help reduce low mood/depression among residents whose identity and occupations have been disrupted by (stroke‐related) disability (Robison et al., 2009). Homes may need to consider some re‐balancing of perceptions of safety and risk regarding residents' participation in valued activities and have explicit conversations with staff, residents and relatives about this.

## AUTHOR CONTRIBUTIONS

JM, FM, CS, CM, IM, CW: Conceptualisation/Methodology. ES: Data curation. ES, SC, JH, CM: Formal analysis. JM, FM, CS, CM, IM, CW: Funding acquisition. ES, SC, JH: Investigation. ES, IM: Project administration. CM, IM: Supervision. ES, SC, JH: Writing—original draft. ES, SC, JH, JM, FM, CS, CM, IM, DW, CW: Writing— review & editing.

## CONFLICT OF INTEREST

All co‐authors confirm that we have no conflict of interest to declare.

## DISCLAIMER

The views expressed are those of the authors and not necessarily those of the funder/supporters.

## Supporting information


Appendix S1

## Data Availability

The data that support the findings of this study are not shared due to the consent agreement with participants.
