# Supplementary material for: The provision of person‐centred care for care home residents with stroke: An ethnographic study
Source: Health Soc Care Community. 2022 Jul 23;30(6):e5186–95. doi: 10.1111/hsc.13936 (PMC10084099; doi:10.1111/hsc.13936)
Supplement: Supplementary file 1 — Appendix S1 [file HSC-30-e5186-s001.docx]

Semi-structured interview guide for care home residents’ friend/family member

Interviewee ID: Care home ID:

Consent form completed

1. Thank you for participating. *Researcher reiterates that interview will be up to 45 minutes [30 more likely], and cover topics around stroke and care needs.*
2. *Introductions*: *confirm relationship to resident if necessary*
3. *History of care and residence*: can you tell me a bit about when [resident] moved into the home?

Prompts:

- What prompted move
- View on whether resident enjoys living at home

1. *History of stroke*: do you recall what happened when [resident] had a stroke? What effects did it have for [resident]?

Prompts:

- How long ago stroke occurred (approximately), where it occurred [confirm with staff]
- Short term vs long term; current effects if any

1. *Multimorbidity*: Does [resident] have other health problems currently? How do they affect [resident]? From your point of view, which is most important for [resident] at the moment (and why e.g. most impact on quality of life)?
2. *Care needs*: What care/help does [resident] need?

Prompts:

- Day-to-day activities? E.g. eating, drinking, getting dressed, leisure/hobbies, going to the toilet
- Medical/health care? E.g. medications, dressings, positioning
- Does [resident] need any special/extra help due to effects of his/her stroke?
- Can [resident] communicate what they need help with when they need it?

1. Which parts of [resident’s] care work well *(check if from their perspective i.e. witnessing care or from what resident has told them)*?

Prompts:

- (CQC aspects of care: effectiveness, responsiveness, feeling cared for, feeling safe, care being well-led)
- Tailored to needs?
- Access to specialists/other health care professionals as needed (GP, nurse, physio etc)
- Information on healthcare and treatment options
- Informed consent / Shared decision making

1. What do you think [resident] finds difficult or dislikes, if anything, about living in the home?

Prompts:

- Daily activities /life in the home
- Care received
- Other difficulties e.g. sleep

1. From your point of view, are there any parts of care/help/accommodation which could be improved?

Semi-structured interview guide for care home residents

Interviewee ID: Care home ID:

Consent form completed

1. Thank you for participating. *Researcher reiterates that interview will be up to 45 minutes, and cover topics around stroke and care needs.*
2. *Introductions*: please can you tell me a bit about yourself?
3. *History of care and residence*: can you tell me a bit about when you moved into this home? Can you tell me your thoughts about living here?
4. *History of stroke*: do you recall having a stroke? What effects did it have on you?

Prompts:

- How long ago stroke occurred (approximately)
- Short term vs long term; current effects if any

1. *Multimorbidity*: Do you have other health problems at the moment? From your point of view, which is most important for you at the moment? (and why?) How do they affect you?
2. Tell me about how you manage with day-to-day activities: (prompts if needed: consider eating, drinking, getting dressed, leisure/hobbies, going to the toilet)

Prompts:

- What care/help is needed?
- Who cares for/helps you e.g. staff, visitors?
- Communicating what you need help with and when you need it

1. Which parts of your care work well from your perspective?

Prompts:

- effectiveness, responsiveness, feeling cared for, feeling safe, care being well-led
- Having relevant information about healthcare and treatment options/shared decision making

1. What do you find most difficult?

Prompts:

- Difficulties with daily activities (see q6) /life in the home
- Difficulties with care received
- Other difficulties e.g. sleep

1. From your point of view, are there any parts of care/help/accommodation which could be improved?

Semi-structured interview guide for care and nursing staff/care home managers

Care staff / Nursing staff / Clinical manager / Operational manager (circle)

Interviewee ID: Care home ID:

Consent form completed

1. Thank you for participating. *Researcher reiterates that interview will be up to 45 minutes, and cover topics around care for residents who have had a stroke.*
2. Introductions: please can you tell me a bit about your…?

Prompts:

- Professional background: length of time in care profession & in current role
- Qualifications and where gained (UK?)
- Current responsibilities
- Other relevant experience

1. Experiences in this care home: Can you tell me your thoughts about working here?

Prompts:

- Views on the home: initial and current impressions of working environment/culture, leadership

1. About the home (manager)

- Number of beds? At capacity?
- All beds same level of care?
- Palliative care provision
- Staff: minimum qualifications/experience requirements

1. Can you tell me about what training you have had to do your current role?

Prompts (brief details):

- specific conditions e.g. dementia
- lifting/ handling
- general e.g. safeguarding, medicines
- Training perceived as up to date?
- Further training needs? How are these identified?
- Barriers to training receipt/implementation

1. Care records

- When are care records updated e.g. updated after health professional visit? Who updates?
- Advance statements/decisions: do many residents have these? Residents with stroke in particular? Have they ever been (needed to be) acted on?

1. Visiting professionals

- GP for home, or GP per resident? Regular visits, or as needed?
- Social workers?
- District nurses, therapists, other?
- Aware who to contact for referrals or advice (health, mental health)? Are other agencies responsive?

1. Can you tell me what you understand about what a stroke is?

*(For those who have awareness of stroke, following questions about experience of working with residents with stroke. If participant has no awareness of what stroke is, then A. have a discussion about what stroke is, B. ask the questions about residents in general, and C. as a follow up question, ask how they believe stroke might affect)*

1. Are you aware of any residents who have had a stroke? In your experience, what particular needs do residents have after stroke?

Prompts:

- Continence, swallowing, ADL, emotional/psychological, medical needs (e.g. risk factor management), positioning, pain, communication aids, vision

Probes:

- What sort of things do you have to help them with?
- How needs assessed/addressed (& by whom): any system to classify stroke re impairment/activity? protocols/policies; workarounds
- Where do you/would you seek advice on stroke care?
- Awareness of clinical guidelines
- Views on aims of relevant care e.g. reducing dependency, quality of life
- Is there anything you need to do to prevent a recurrent stroke?
- (if residents on anticoagulant e.g. warfarin or novel ones, or antiplatelet e.g. aspirin, clopidogrel) Awareness of anticoagulation/antiplatelet effects re risk of bleeding etc

1. Is there anything which makes caring for residents difficult? And in those who have had a stroke in particular?

Prompts:

- Lifting/handling
- Time pressure/staffing safe levels
- Training
- Other challenges: continuity of care, facilities/equipment
- Care for residents with co-morbidities e.g. dementia
- Changes in challenges over time/in their experience

1. From your perspective, what would help improve the care of people after stroke in care homes?
2. Is there anything else you would like to mention in relation to caring for residents with stroke?
